# T Follicular Helper Cells in Tertiary Lymphoid Structure Contribute to Renal Fibrosis by IL-21

**DOI:** 10.3390/ijms241612535

**Published:** 2023-08-08

**Authors:** Ran Luo, Dan Chang, Nanhui Zhang, Yichun Cheng, Shuwang Ge, Gang Xu

**Affiliations:** Department of Nephrology, Tongji Hospital, Tongji Medical College, Huazhong University of Science and Technology, Wuhan 430030, China; ranluo@tjh.tjmu.edu.cn (R.L.);

**Keywords:** T follicular helper cells, tertiary lymphoid structure, renal fibrosis, IL-21

## Abstract

Tertiary lymphoid structure (TLS) represents lymphocyte clusters in non-lymphoid organs. The formation and maintenance of TLS are dependent on follicular helper T (TFH) cells. However, the role of TFH cells during renal TLS formation and the renal fibrotic process has not been comprehensively elucidated in chronic kidney disease. Here, we detected the circulating TFH cells from 57 IgAN patients and found that the frequency of TFH cells was increased in IgA nephropathy patients with renal TLS and also increased in renal tissues from the ischemic-reperfusion-injury (IRI)-induced TLS model. The inducible T-cell co-stimulator (ICOS) is one of the surface marker molecules of TFH. Remarkably, the application of an ICOS-neutralizing antibody effectively prevented the upregulation of TFH cells and expression of its canonical functional mediator IL-21, and also reduced renal TLS formation and renal fibrosis in IRI mice in vivo. In the study of this mechanism, we found that recombinant IL-21 could directly promote renal fibrosis and the expression of p65. Furthermore, BAY 11-7085, a p65 selective inhibitor, could effectively alleviate the profibrotic effect induced by IL-21 stimulation. Our results together suggested that TFH cells contribute to TLS formation and renal fibrosis by IL-21. Targeting the ICOS-signaling pathway network could reduce TFH cell infiltration and alleviate renal fibrosis.

## 1. Introduction

Despite a massive amount of research inquiring into the relationship between immunological processes and the development of renal fibrosis, there are still many unsolved questions around chronic kidney disease (CKD) [1]. Tertiary lymphoid structure (TLS) represents a form of ectopic, highly organized collections of T cells and B cells with a specific structure similar to the secondary lymphoid organ (SLO). Although the inducing conditions of TLS and their contribution to disease pathology are not fully understood, it seems to play a crucial role in local antigen presentation and immune activation in chronic inflammation, including Heliobacter pylori infection and Mycobacterium tuberculosis [2]. In addition, TLSs are likely to initiate or support the production of self-reactive antibodies and B cell survival in autoimmune diseases, including Hashimoto thyroiditis, multiple sclerosis, diabetes, and rheumatoid arthritis [3]. Recently, TLS has been recognized to be associated with chronic kidney disease progression [4] and acute renal allograft rejection [5]. TLS-associated affairs during CKD progression include the upregulation of inflammatory cytokines and recruitment of various immune cells, especially CD4+ T cells and B cells in the kidney [6]. The gene expression profile of renal TLS closely resembled lymph nodes, indicating that TLS may have a functional germinal center (GC) and participate in the disease’s development [7].

Follicular helper T (TFH) cells are a special subpopulation of effector CD4+ T cells, which are most commonly recognized as CXCR5+CD4+ cells and also express inducible T-cell co-stimulator (ICOS) molecule and the coinhibitory molecule programmed cell death protein 1 (PD-1) [8]. In the GC of SLO, successful interactions with TFH cells promote antigen-specific B cell affinity maturation and further differentiation [9]. TFH cells are critical for generating and maintaining germinal centers in SLO [9]. In addition to lymph nodes and spleen, TFH cells were identified in non-lymphoid tissues, especially in immune-associated diseases, including infection, autoimmune diseases, and cancer [10]. Although the orchestration of the lymphotoxin and chemokine seems to be critical for TLS formation, TFH cells are also indispensable for the formation and maintenance of germinal centers of TLS [11]. TFH cells migrate to B cell follicles under the action of CXCR5 ligand chemokine CXCL13, and then regulate the differentiation of B cells through ICOS–ICOS ligand [9]. However, the potential role of TFH cells during renal TLS formation and renal fibrotic process is still unclear.

IL-21, a canonical functional mediator of TFH cells, is often observed in chronically inflamed tissue, such as lupus nephritis, Sjogren’s syndrome, and atherosclerosis [12]. IL-21 neutralization inhibits GC maturation, reduces autoantibody production, delays the progression of glomerulonephritis, and improves overall survival in lupus-prone mice [13]. Furthermore, through interacting with differentiated fibroblasts, TFH cells are engaged in both the early inflammatory and late fibrotic phases by IL-21 [14]. IL-21 neutralization is reported to prevent skin fibrosis in mice with graft-versus-host disease [15]. In addition, the expression of IL-21 receptor (IL-21R) is detected in infiltrating lymphocytes of human pulmonary fibrosis specimens, suggesting that IL-21-responsive lymphocytes might be related to the progression of pulmonary fibrosis [16]. IL-21R deficiency significantly attenuated the progression of liver fibrosis in parasitic infection mice [17]. However, the underlying mechanisms of IL-21 and renal fibrosis remain poorly defined.

To evaluate the role of TFH cells and IL-21 in renal TLS formation and fibrosis, we compared the frequency of circulating TFH cells in CKD patients with or without renal TLS. The expressions of IL-21, formation of TLS, and evaluation of renal fibrosis were measured in mouse kidneys after ischemic-reperfusion injury with or without the utilization of ICOS-neutralizing antibodies to inhibit TFH cell differentiation and function. Furthermore, we assessed the role of IL-21 in fibroblast activation and explored the intracellular signaling pathways after IL-21 administration.

## 2. Results

### 2.1. IgAN Patients with Renal TLS Had Increased Frequency of TFH Cells

**Figure 1:**
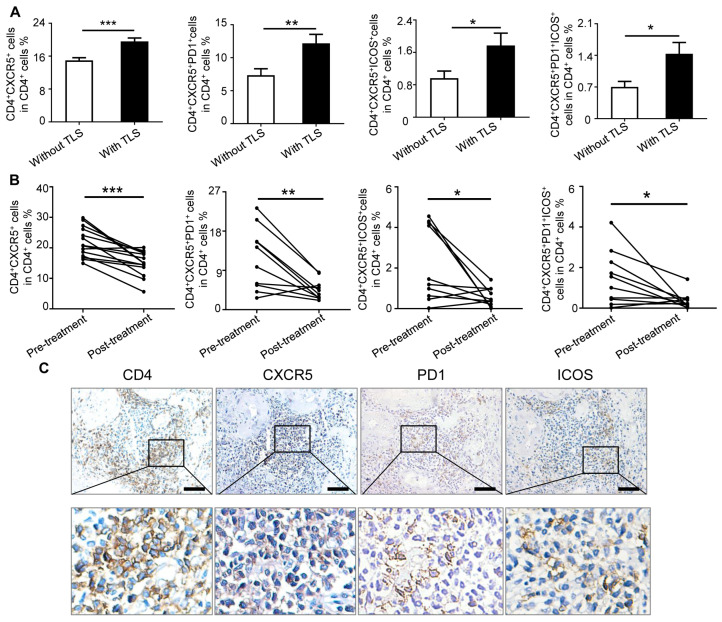
IgAN patients with renal TLS had increased frequency of TFH cells compared to those without renal TLS. Flow cytometry analysis of the percentage of various subsets of TFH cells. PBMCs from IgAN patients were stained with anti-CD4, anti-CXCR5, anti-PD1, and anti-ICOS antibodies. Cells were gated on living lymphocytes and CD4+T cells. (**A**) The quantitative frequency of CD4+CXCR5+ TFH cells, CD4+CXCR5+PD1+ TFH cells, CD4+CXCR5+ICOS+ TFH cells, and CD4+CXCR5+PD1+ICOS+ TFH cells among CD4+ T cells was analyzed by flow cytometry in IgAN patients without renal TLS (*n* = 27), and IgAN patients with renal TLS (*n* = 30). (**B**) The percentages of different subsets of TFH cells were compared in IgAN patients before and after the treatment (*n* = 10). (**C**) The representative immunohistochemical staining of CD4, CXCR5, PD1, and ICOS in renal TLS group. * *p* < 0.05, ** *p* < 0.01, *** *p* < 0.001. Data represent mean ± SEM, Scale bar = 100 μm. *p*-values were calculated using a two-tailed *t*-test.

Previous studies reported that CD4+CXCR5+ TFH cells in the circulation of IgAN patients were increased compared to healthy control [18]. To explore the relationship between renal TLS and circulating TFH cells, we compared the frequency of TFH cells in IgAN patients grouped by renal TLS. The baseline characteristics of the 57 IgAN patients are presented in Table S2. The percentage of CD4+CXCR5+ TFH cells was higher in patients with renal TLS, compared to those without renal TLS (Figure 1A). The frequency of the TFH cells subset, including CD4+CXCR5+PD1+ TFH cells and CD4+CXCR5+ICOS+ TFH cells, was also increased in IgAN patients with renal TLS formation (Figure 1A). Next, to further evaluate the clinical significance of TFH cells, we collected and analyzed the frequency of TFH cells after immunosuppression treatment in 10 patients followed up for 12 weeks; all of the 10 patients at least received corticosteroids immunotherapy treatment (Table S3). After treatment, the frequency of CD4+CXCR5+ TFH, CD4+CXCR5+PD-1+ TFH cells, and CD4+CXCR5+ICOS+ TFH cells, was dramatically decreased (Figure 1B). The level of proteinuria, blood urea nitrogen, and uric acid declined after immunotherapy treatment (Table S4). To locate TFH cells in renal tissue, TFH cell biomarkers of CD4, CXCR5, PD1, and ICOS were stained by immunohistochemistry (IHC). The results of serial section staining showed that TFH cells were mainly aggregated in renal TLS of IgAN patients (Figure 1C).

### 2.2. TFH Cells Were Increased in IRI-Induced Renal TLS Mice

**Figure 2:**
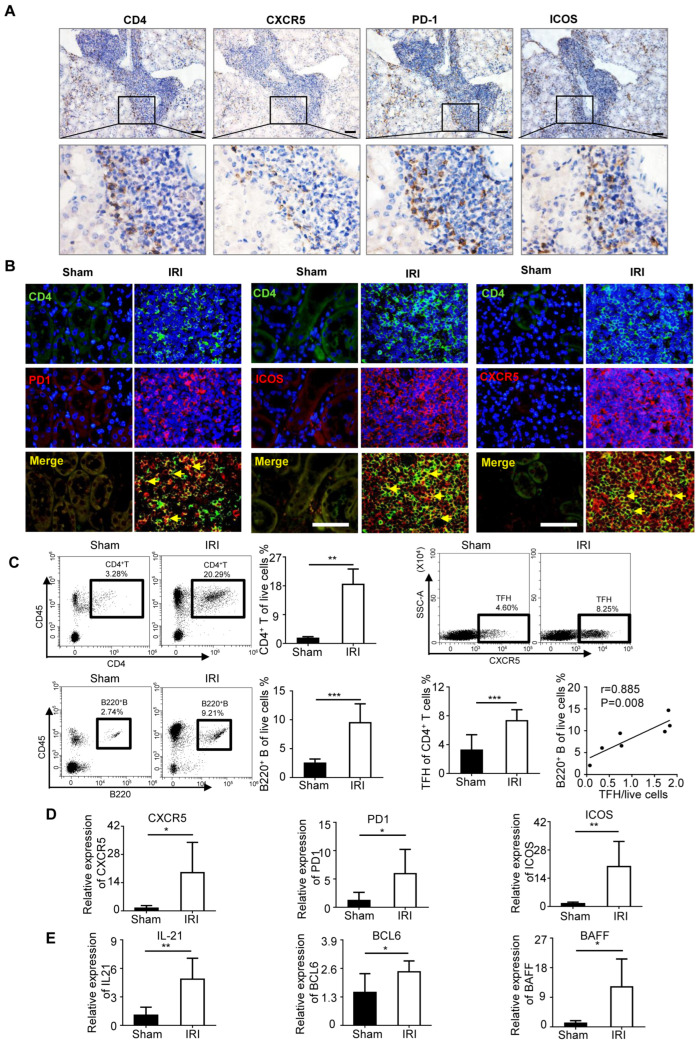
TFH cells were upregulated in IRI-induced renal TLS mice. (**A**) Representative IHC staining of CD4, CXCR5, PD1, and ICOS in IRI-induced renal TLS sample. (**B**) Representative immunofluorescence staining of CD4+ PD1+, CD4+ ICOS+, CD4+ CXCR5+ TFH infiltrating cells in TLS kidney. Yellow arrows indicate positive cells. (**C**) The percentage of CD4+ T cells, CD4+CXCR5+ TFH cells, and B220+ B cells was analyzed by flow cytometry from the whole kidney in the sham group and TLS group (*n* = 6 per group). Real-time PCR analysis of TFH cells phenotype (**D**) and B cell-related (**E**) markers in kidneys of the sham group and TLS group (*n* = 6 per group). * *p* < 0.05, ** *p* < 0.01, *** *p* < 0.001. Data represent mean ± SEM, Scale bar = 50 μm. *p*-values were calculated using a two-tailed *t*-test.

To investigate the frequency of TFH cells in the kidney, we established TLS mice models by IRI as previously reported. The phenotype of TFH cells was further processed by IHC and IF labeling. The IHC staining verified that the existence of CXCR5, PD-1, and ICOS single-positive cells was mainly localized in the TLS area (Figure 2A). IF staining revealed that CXCR5, PD-1, and ICOS were co-localized with CD4+ cells (Figure 2B). FACS results showed that the CD4+CXCR5+ TFH cells were expanded markedly in the kidney of the IRI group (Figure 2C). Meanwhile, TFH cell expression was intensely positively correlated with B220+ cells (Figure 2C). The mRNA expression of TFH phenotype markers was heightened in the IRI kidney (Figure 2D). Additionally, the expressions of BCL-6, IL-21, and BAFF were also increased in the kidney of IRI mice (Figure 2E). These data indicated the necessity of TFH cell infiltration in the TLS model.

### 2.3. Anti-ICOS Depletion Restrained the TLS Formation

**Figure 3:**
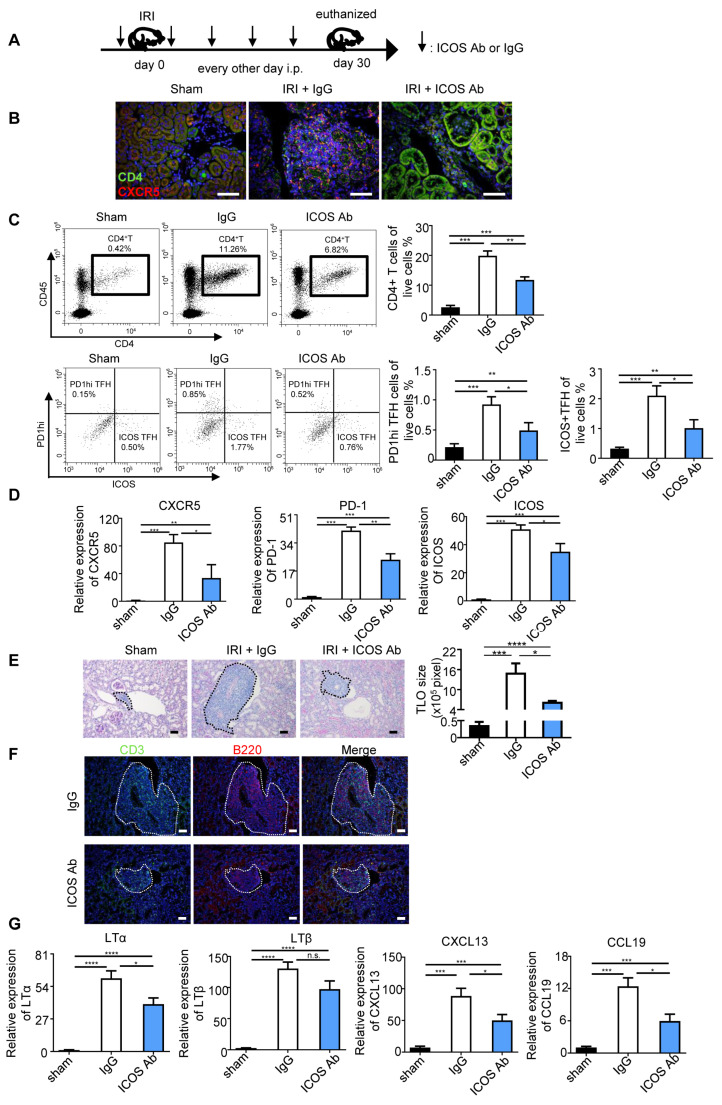
Blocked-off ICOS restrained the TLS formation. (**A**) Scheme. Aged mice were treated intraperitoneally with either 250 μg of anti-ICOS antibody or IgG at day 1 before renal IRI, followed by every other day (*n* = 5 per group). (**B**) Representative IF staining of CXCR5+CD4+ TFH cells in each group. (**C**) The percentage of CD4+ T cells, PD1hi TFH cells, and ICOS+ TFH cells was analyzed by flow cytometry from the whole kidney in the IgG and anti-ICOS antibody treatment groups. (**D**) Real-time PCR analysis of CXCR5, PD1, and ICOS expression in the kidney. (**E**) Representative photomicrographs and statistical graphs for PAS staining of TLS size. (**F**) Immunofluorescence analysis of CD3 and B220. (**G**) Real-time PCR analysis of lymphotoxin and chemokines expression in the kidney. n.s. means no significant, * *p* < 0.05, ** *p* < 0.01, *** *p* < 0.001, **** *p* < 0.0001. Data represent mean ± SEM, Scale bar = 50 μm. *p*-values were calculated using a two-tailed *t*-test.

Signals through ICOS and its ligand are critical for TFH cell differentiation and maintenance [19]. To explore the link between TFH cells and disease pathogenesis, we applied an ICOS neutralization antibody to block TFH cells in the IRI-induced TLS mice (Figure 3A). As shown in Figure 3B, the co-staining intensity of CD4 and CXCR5 was reduced in the ICOS antibody group. Consistently, administration of the ICOS antibody significantly inhibited the elevation of CD4+ T cells, PD-1hiCXCR5+CD4+ TFH cells, and ICOS+CXCR5+CD4+ TFH cells in the kidney from IRI mice compared to the IgG group (Figure 3C). The mRNA expression levels of TFH cell membrane markers, including CXCR5, PD-1, and ICOS, were consistently reduced by the ICOS neutralization antibody (Figure 3D). Administration of the ICOS antibody effectively reduced the infiltrating TFH cells in the kidney. Further, PAS and IF staining on renal sections were performed to characterize TLS formation. As shown in Figure 3E, the size of the TLS significantly decreased in the ICOS antibody-treated group. Meanwhile, IF staining analysis also verified the diminishing of CD3+ T cells and B220+ B cells in the renal TLS of anti-ICOS-treated mice (Figure 3F), implicating an extraordinary inhibiting effect in TLS construction of the ICOS blockade. Lymphotoxin (LTα and LTβ) and chemokine (CXCL13 and CCL19) are crucial cytokines in TLS formation. Therefore, we also evaluated their expression and found that both lymphotoxin and chemokines were highly expressed in IRI-induced renal TLS injected with IgG, whereas their expression was reduced by ICOS-neutralizing antibody treatment (Figure 3G). These results indicated that TFH facilitated pathological renal TLS formation in the IRI-induced mice model.

### 2.4. Anti-ICOS Depletion Depressed the Production of IL-21 and Ameliorated Fibrosis

**Figure 4:**
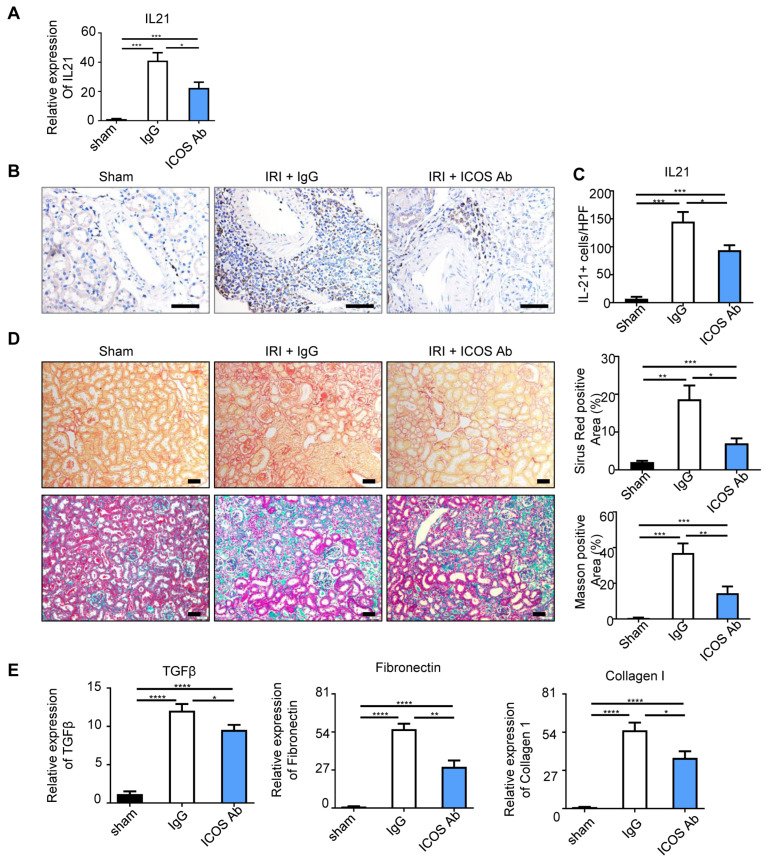
Anti-ICOS depletion depressed the production of IL-21 and ameliorated fibrosis. (**A**) Real-time PCR analysis showed the RNA levels of IL-21 in sham, IRI + IgG, and IRI+ ICOS Ab group. Representative images (**B**) and quantitative analysis (**C**) of renal IL-21 positive cells in each group. (**D**) Representative photomicrographs of collagen staining by Sirius red and Masson blue, and the percentage of Sirius red and Masson blue in each group. (**E**) Real-time PCR results showed mRNA expressions of TGF-β, fibronectin, and collagen I in each group. *n* = 5 per group. * *p* < 0.05, ** *p* < 0.01, *** *p* < 0.001, **** *p* < 0.0001. Data represent mean ± SEM, Scale bar = 50 μm. *p*-values were calculated using a two-tailed *t*-test.

TFH cell-derived Interleukin-21 (IL-21), is a crucial factor for B-cell maturation and has been conjoined to fibrosis [20]. The mRNA level of IL-21 was markedly elevated (*p* < 0.005) in the kidney from the IRI-induced TLS mice model and partially restrained by ICOS neutralization antibody treatment (Figure 4A). Likewise, the IHC staining showed that IL-21 was dispersed conspicuously at the TLS area, where the TFH cells were mostly aggregated (Figure 4B). In any case, the elevated expression of IL-21 was suppressed by ICOS neutralization antibody treatment (Figure 4C).

To assess the impact of the depletion of TFH cells on fibrosis, we evaluated renal sections from the different treatment animal groups by Masson and Sirius red staining (Figure 4D). Anti-ICOS depletion led to essentially diminished collagen deposition of the kidney. Other profibrotic factors were also detected and qPCR data showed that TGF-β, fibronectin, and collagen I were also increased in the isotype-injected IRI kidney and reduced after ICOS neutralization antibody treatment (Figure 4E).

**Figure 5:**
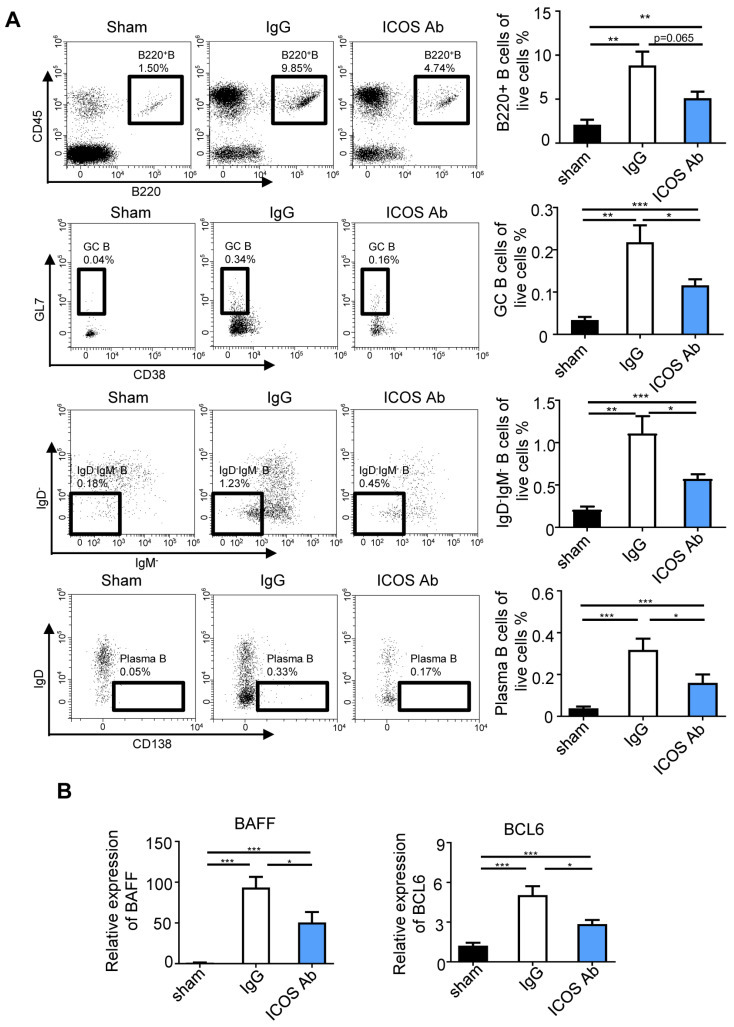
The differentiation of B cells was altered after ICOS-antibody treatment. (**A**) The percentages of B220+ B cells, GC B cells, IgD-IgM- B cells, and plasma B cells were analyzed by flow cytometry from the whole kidney in the IgG and anti-ICOS antibody treatment groups. (**B**) Real-time PCR analysis revealed upregulation of BAFF and BCL-6 expressions in kidney was abolished by anti-ICOS antibody treatment. *n* = 5 per group. * *p* < 0.05, ** *p* < 0.01, *** *p* < 0.001. Data represent mean ± SEM. *p*-values were calculated using a two-tailed *t*-test.

We conducted FACS analysis of B cell subsets isolated from IRI kidneys to further investigate the effects of ICOS neutralization antibody treatment. Compared with the isotype-treated group, the total of B220+ cells in the ICOS-neutralizing antibody treatment group showed a decreasing trend. Notably, anti-ICOS depletion effectively inhibited the expansion of GC+ B cells, IgD-IgM- B cells, and plasma B cells in the IRI-induced renal TLS model (Figure 5A). The expression of BAFF and BCL6, which were critical for B cell growth and proliferation, was dramatically upregulated in the kidney from isotype-treated IRI mice and was abrogated by ICOS neutralization antibody treatment (Figure 5B).

Together, these results demonstrated that the anti-ICOS depletion of TFH cells could powerfully restrict renal TLS formation and protect against fibrosis. In addition, class switching of B cells in germinal centers was also inhibited by ICOS-neutralizing antibody treatment in vivo.

### 2.5. IL-21 Directly Promoted Renal Fibrosis through Activating NF-κB Pathway

**Figure 6:**
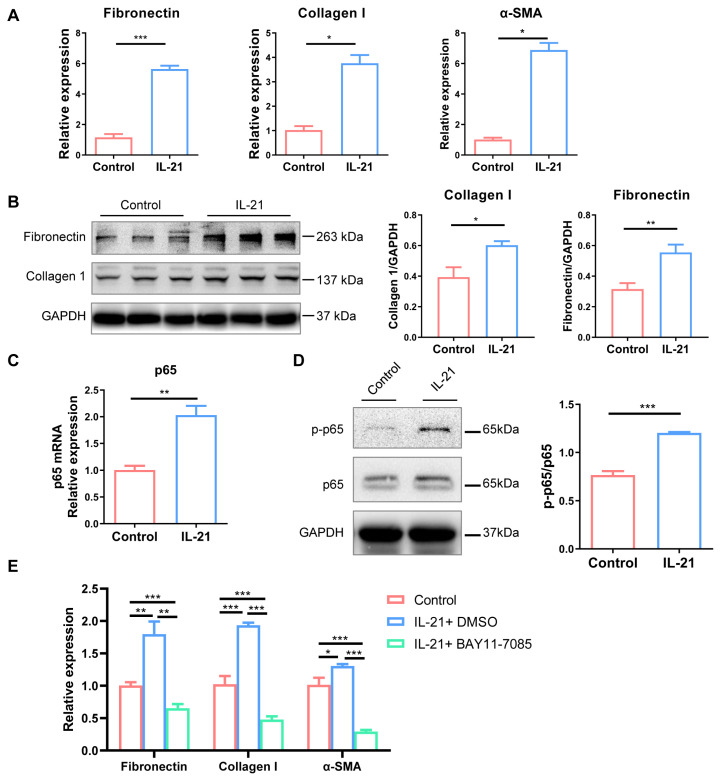
IL-21 directly promoted renal fibrosis through activating NF-κB pathway. NRK-49F cells were stimulated with recombinant IL-21 (100 ng/mL) for 48 h. (**A**) The mRNA expression level of fibronectin, collagen I, α-SMA, and RelA in the two groups. (**B**) Western blot was used to measure the expression of fibronectin and collagen I in the two groups. (**C**) The mRNA expression of p65 in the two groups. (**D**) Western blot was used to measure the expression of p-p65 and p65 in the two groups. (**E**) Real-time PCR analysis of the expression of fibronectin, collagen I, and α-SMA expressions in IL-21 stimulated NRK-49F cells with or without p65 inhibitor BAY 11-7085. * *p* < 0.05, ** *p* < 0.01, *** *p* < 0.001. *p*-values were calculated using a two-tailed *t*-test.

To evaluate the influence of IL-21 as a pro-fibrotic cytokine on renal fibrosis, we stimulated NRK-49F cells with recombinant IL-21. The qPCR (Figure 6A) and Western Blot (Figure 6B) analysis showed that IL-21 stimulation directly induced significantly increased mRNA and protein expression of extracellular matrix protein, including fibronectin and collagen I. Meanwhile, the expression of p65, a vital transcription factor of the NF-κB pathway, was increased after IL-21 stimulation (Figure 6C). Western blot analysis showed that phosphorylation of p65 in NRK-49F cells was increased after recombinant IL-21 stimulation (Figure 6D). Furthermore, BAY 11-7085, a p65 selective inhibitor, could effectively alleviate the pro-fibrotic effect induced by IL-21 stimulation (Figure 6E). These results further emphasized the critical role of IL-21-mediated pro-fibrotic effects in renal fibrosis through activating the NF-κB pathway.

## 3. Discussion

Our study indicated that TFH cells in TLS contributed to the pathogenesis of renal fibrosis in CKD. In the present study, we showed that renal TFH cells were mainly aggregated in the TLS area. Furthermore, the frequency of TFH cells was upregulated in IgAN patients with renal TLS and in the IRI mice model. Of note, TFH cells’ attenuation by the ICOS neutralization antibody resulted in the inhibition of TLS formation, IL-21 expression, and renal fibrosis in vivo. Finally, we showed that IL-21 directly promoted fibrosis in vitro.

In our study, we demonstrated that TFH cells were accumulated in the TLS kidney of patients with chronic kidney disease. Elevated TFH cells were accompanied by increased CXCL13 expression in mice subjected to ischemia-reperfusion injury. Chemokine CXCL13 is primarily generated by lymphoid tissue organizer cells and plays a vital role in lymphoid organization, lymphoid regeneration, and immune response [21]. Recently, CXCL13 chemokine was reported to be associated with the pathogenesis of various autoimmune diseases (for instance, primary Sjogren syndrome, rheumatoid arthritis, multiple sclerosis, and systemic lupus erythematosus) [21]. It was reported that local and serum levels of CXCL13 were upregulated in unilateral and bilateral renal IRI mouse models, which is in agreement with our current results [22]. Functionally, CXCL13 binds to its typical ligand of CXCR5 expressed on TFH cells and may play a potential role in TFH cell recruitment. Indeed, IRI-induced TLS was smaller in CXCL13-deficient mice, suggesting that CXCL13 represents a critical factor in renal TLS formation.

Remarkably, it is reported that circulating TFH cells exhibit nearly similar phenotypic and functional characteristics of TFH cells in the germinal center of lymph nodes [23]. In accordance with previous studies, we found that the rising trend of TFH cells frequency in the spleen was similar in the animal kidney. Furthermore, we demonstrated that the frequency of TFH cells in the peripheral blood of IgAN patients was correlated with TLS numbers in the fibrotic kidney. TFH cells circulate between secondary lymphoid organs and blood [24], indicating that TFH cells in the peripheral blood of IgAN patients may reflect abnormal immune responses in renal interstitial tissues. In addition to IgA nephropathy, activated circulating TFH cells were correlated with the disease severity of patients with lupus nephritis [25], membranous nephropathy [26], and kidney transplantation [27]. Immunotherapies, including corticosteroids, are known to inhibit the production of a large number of cytokines and inhibit the T/B cells response. In this study, the percentage of circulating TFH cells decreased significantly after immunotherapy treatment of IgAN patients; this result is consistent with previously reported studies among patients with rheumatoid arthritis [28]. Concurrently, the improvement of clinical symptoms after treatment was observed. The levels of proteinuria, blood urea nitrogen, and uric acid were diminished after immunotherapy treatment. These data indicated that the percentage of circulating TFH cells was a potential marker of disease severity. Larger sample sizes and longer follow-up cohorts are needed to prove their effectiveness.

ICOS and its ligand interaction induce differentiation and maintenance of TFH cells, not that ICOS expression by TFH cells is necessary for GC formation [29]. ICOS loss was found in patients with variable immunodeficiency, accompanied by a decrease in TFH cells [30]. Accumulation of TFH cells was increased in Roquin mutant mice that were unable to degrade ICOS [31]. ICOS upregulation is relevant to disease activity in a variety of inflammatory diseases, such as inflammatory bowel disease [32] and asthma [33]. To specifically deplete TFH cells, restraint of ICOS/ICOS-L interactions has been widely used in various mouse models [34]. Indeed, we showed that administration of the ICOS antibody effectively reduced the infiltrating TFH cells in the kidney with decreased tissue fibrosis, implicating an extraordinary effect of ICOS and its ligand receptor-ligand in renal TFH development. The reduced TLS-associated chemokines and cytokines in the kidney after ICOS inhibition demonstrated the essential role of ICOS signaling in renal TLS formation. In addition, the anti-ICOSL monoclonal antibody pre-treatment has a therapeutic effect on joint inflammation in systemic lupus erythematosus patients [35], indicating that ICOS/ICOS-L blockade may be therapeutically beneficial in renal intestinal inflammation.

TFH cells can promote kidney fibrosis through a variety of mechanisms. Firstly, TFH cells can promote the recruitment of other immune cells and maintain the structure and function of TLS, thus contributing to the chronic inflammatory response [36]. In our study, the infiltration of renal TFH cells was implicated in the expression of chemokines and accumulation of inflammatory cells that helped to maintain the local inflammatory microenvironment and recruit other immune pathogenic cells. Secondly, it is reported that TFH cell-associated IL-21 could combine with IL-21R on fibroblasts in the gut and upregulate matrix metalloproteinase expression, raising the possibility that IL-21 could act directly on fibroblasts [37]. Besides, CD4+ IL-21+ cells sorted from the synovial fluid can induce fibroblast-like synovial cells to secrete MMP-1 and MMP-3, which promote inflammation and joint pathological changes in patients with rheumatoid arthritis [38]. In the graft-versus-host disease mice model, IL-21-neutralizing antibodies block the pathological progression of the disease by reducing skin fibrosis [15]. In this study, IL-21 could upregulate the expression of fibronectin and collagen in rat renal kidney fibroblast cells (NRK-49F), which were blocked by NF-κB inhibitor BAY 11-7085. Finally, other fibro-genic factors, represented by TGF-β1, were elevated in the kidney and were decreased after TFH inhibition by the ICOS antibody, which further indicated that TFH cells had a role in promoting fibrosis in the kidney.

There are some limitations of the present study that should be mentioned. First, the number of patients in this study was small and may have been limited by low statistical power. Secondly, the limited size of the patient kidney biopsy tissue makes it difficult to isolate human renal T cells for FASC. Third, the mouse kidney fibrosis model after ischemia-reperfusion injury in this study, does not perfectly phenocopy the human chronic kidney disease. Finally, the use of ICOS antibodies does not specifically target TFH cells. However, there are no approved therapeutic agents that target TFH cells to assess their direct effect on disease progression. Alleviation of the inflammatory and fibrotic phenotype after treatment with an anti-ICOS monoclonal antibody in our study revealed the pathogenic role of TFH in renal TLS formation and fibrosis.

In conclusion, our data show that T follicular helper cells contribute to TLS formation and renal fibrosis by IL-21. Targeting the ICOS-signaling pathway network could reduce TFH cell infiltration and alleviate renal fibrosis.

## 4. Materials and Methods

### 4.1. Ethics Approval of Participants and Animals

Ethical Committee of Huazhong University of Science and Technology approved the study, approved protocol #TJ-IRB20210722. Laboratory Animal Centre, Huazhong Agriculture University approved all animal experiments, and approved protocol #HZAUMO-2021-0153. C57BL/6J mice (10–12 months, 30–35 g) were purchased from Vital River (Beijing, China) and housed in Laboratory Animal Centre, Huazhong Agriculture University with a specific pathogen-free condition. Mice were euthanatized by carbon dioxide and samples were collected.

### 4.2. Renal Tertiary Lymphoid Structure (TLS) Model

For the induction of renal TLS, we used ischemic-reperfusion injury (IRI) models, as previously reported [4]. Briefly, the left kidneys were clamped with an atraumatic vascular clip (Roboz Surgical Instrument Co., Stuttgart, Germany) for 37 min after anesthesia. IRI mice were euthanized 30 days after the surgical procedure. Age-matched male mice were subjected to a similar operation without clamping as the sham group. For blocking TFH, an anti-ICOS antibody or IgG (Bio X Cell, West Lebanon, Lebanon, NH, USA, 200 μg) was injected intraperitoneally one day before and every other day after the IRI operation until euthanized.

### 4.3. Flow Cytometry of Analysis

The kidney tissue was minced with sterile scissors and digested with 1 mg/mL collagenase I for 45 min. Tissue suspension was terminated by digestion with PBS, filtrated by a strainer, and centrifuged at 1500 rpm for 5min. A single-cell suspension was prepared after red blood cell lysis. Cells were counted with a hemocytometer, and cell concentration was adjusted according to the count so that the cell suspension of each tube was 1 × 10^6^ cells. These samples were incubated with antibodies from Biolegend (San Diego, CA, USA) against BV510-conjugated Zombie, BV421-conjugated anti-mouse CD4 antibody, APC/Cy7-conjugated anti-mouse CD45 antibody, PE-conjugated anti-mouse PD1 antibody, PE/Cy7-conjugated anti-mouse CXCR5 antibody, BV605-conjugated anti-mouse ICOS antibody, PE-conjugated anti-mouse B220 antibody, Pecy5.5-conjugated anti-mouse IgD antibody, PE/Cy7-conjugated anti-mouse IgM antibody, FITC-conjugated anti-mouse CD38 antibody, APC-conjugated anti-mouse GL7 antibody, BV421-conjugated anti-mouse CD138 antibody. To detect TFH cells in peripheral blood leukocytes, we used the following antibodies from BD biosciences: KO525-conjugated anti-human CD4 antibody, FITC-conjugated anti-human CXCR5 antibody, PB450-conjugated anti-human PD1 antibody, PE/Cy7-conjugated anti-human ICOS antibody. Scheme of cell sorting for TFH and B lymphocyte subsets is presented in Figures S1 and S2.

### 4.4. Renal Histological Evaluation

Kidneys were fixed with 4% formalin, embedded in paraffin, and cut into 4 um serial slices. Detection of the size of renal TLS was achieved using Periodic acid–Schiff (PAS) staining on kidney sections. As previously reported [4], the definition of TLS size was the accumulation of the TLS in the renal cortex measured by ImageJ software V1.52. Masson and Sirius red staining were used to evaluate renal fibrosis. Image-Pro Plus 6.0 software was used to quantify the positive area.

### 4.5. Immunohistochemistry and Immunofluorescence

Kidneys were fixed with 4% formalin, embedded in paraffin, and cut into 4 um serial slices. After deparaffinization and rehydration, Citrate buffer (pH 6.0) or Ethylene Diamine Tetraacetic Acid (EDTA, pH 9.0) was used for antigen retrieval. For immunohistochemistry staining, after being blocked with 10% H_2_O_2_ for 15 min and 5% serum for 30 min at room temperature, the paraffin sections were incubated at 4 °C overnight with primary antibodies. The primary antibodies included anti-IL-21 (ABclonal, Wuhan, China), anti-CXCR5 (Bioss, Beijing, China), anti-ICOS (eBioscience, San Diego, CA, USA), anti-PD1 antibodies (CST, Framingham, MA, USA). Slices were incubated with horseradish peroxidase (HRP)-conjugated secondary antibodies at room temperature. Then, 3,3′-diaminobenzidine (DAB) was visualized. For immunofluorescence (IF) staining, paraffin sections were blocked with 5% serum at room temperature for 30 min, and then incubated with primary antibodies at 4 ℃ overnight. The primary antibodies included anti-CD4 (Biolegend, San Diego, CA, USA), anti-PD1 (CST, Framingham, MA, USA), anti-CXCR5 (Bioss, Beijing, China), anti-ICOS (eBioscience). Slices were incubated with fluorescence-labeled secondary antibodies and developed with 4′,6-diamidino-2-phenylindole (DAPI). Images were captured using an Olympus microscope.

### 4.6. Real-Time PCR

The total RNAs of kidneys were extracted with Trizol and converted into template cDNA with a reverse transcriptase kit (Vazyme, Nanjing, China). Quantitative polymerase chain reaction (qPCR) was performed with 5 μL the 2× SYBR master-mix (Vazyme, Nanjing, China), 1 μL cDNA, and 10 μmol/L of the primer pairs in a total volume of 10 μL. The primers are listed in Table S1. The expression mRNA level was normalized by GAPDH. The qPCR amplification protocol was according to the manufacturer’s instructions.

### 4.7. Cell Culture and Treatment

Rat renal kidney fibroblast cells (NRK-49F) cells were cultured as previously described [39]. After pre-incubation in serum-free medium for 8 h, the cells were treated with recombinant human IL-21 (100 ng/mL, Peprotech, Waltham, MA, USA) or PBS for 48 h. BAY 11-7085 is a well-known inhibitor of IκBα that leads to the blockage of NF-κB pathway. NRK-49F cells were pre-treated for blocking experiments with BAY 11-7085 1 h. before stimulated with IL-21.

### 4.8. Western Blotting

The isolation of total protein, cytoplasmic protein, and nuclear protein has been described in several previous publications [40,41]. Equal amounts of proteins (25 μg) were separated by 10% SDS-PAGE and transferred to PVDF membranes. The membranes were blocked with non-fat milk (5%) at room temperature for 1 h, and then incubated with primary antibodies against fibronectin (proteintech, Wuhan, China), collagen I (Boster, Wuhan, China), α-SMA (Abcam, Waltham, MA, USA), GAPDH (ABclonal, Wuhan, China), p65 (CST, Danvers, MA, USA), and p-p65 (CST, Danvers, MA, USA) at 4 °C overnight. After being washed with TBST buffer three times, the membranes were incubated with an HRP-conjugated secondary antibody and detected by enhanced chemiluminescence (ECL, BioRad, Hercules, CA, USA). The density of the bands was quantified using ImageJ software V1.52 (US National Institutes of Health, Bethesda, MD, USA).

### 4.9. Statistical Analysis

SPSS 23.0 software (SPSS, Chicago, IL, USA) and GraphPad Prism V8.0.2 (Graph software, San Diego, CA, USA) were used for statistical analysis. Data were presented as mean ± SEM. Correlations were evaluated using parametric Pearson’s correlation tests. One-way ANOVA test, Mann–Whitney test, or unpaired *t*-test were applied to evaluate *p* values as suitable.

## Data Availability

The data underlying this article will be shared upon reasonable request by the corresponding author.

## Supplementary Materials

The following supporting information can be downloaded at: https://www.mdpi.com/article/10.3390/ijms241612535/s1.
