# Supplementary material for: T Follicular Helper Cells in Tertiary Lymphoid Structure Contribute to Renal Fibrosis by IL-21
Source: Int J Mol Sci. 2023 Aug 8;24(16):12535. doi: 10.3390/ijms241612535 (PMC10454845; doi:10.3390/ijms241612535)
Supplement: Supplementary file 1 [file ijms-24-12535-s001.zip › ijms-2489055-supplementary.pdf]

Table S1. Primers used to amplify cDNA for mice.

Primers	Sequence (Sense/Antisense)
CXCR5	5′-ACTCCTTACCACAGTGCACCTT-3′
	5′-GGAAACGGGAGGTGAACCA-3′
PD-1	5′-GGAGCAGAGCTCGTGGTAAC-3′
	5′-GCTGTGAGACTTCCTCCTCG-3′
ICOS	5′-ACTTGCAGGTGTGACCTCAT-3′
	5′-CACCAGCAGAATGTTGTCTGTAG-3′
IL-21	5′-AGCCATCAAACCCTGGAAACA-3′
	5′-TCACAGGAAGGGCATTTAGCTATG-3′
BCL6	5′-CCGGCACGCTAGTGATGTT-3′
	5′-TGTCTTATGGGCTCTAAACTGCT-3′
BAFF	5′-TACCGAGGTTCAGCAACACC-3′
	5′-CCGGTGTCAGGAGTTTGACT-3′
LTα	5′-CCCATCCACTCCCTCAGAAG-3′
	5′-CATGTCGGAGAAAGGCACGAT-3′
LTβ	5′-GAGACAGTCACACCTGTTG-3′
	5′-CCTGTAGTCCACCATGTCG-3′
CXCL13	5′-CGTGCCAAATGGTTACAAAGATT-3′
	5′-GTGGCTTCAGGCAGATCTTC-3′
CCL19	5′-CCTGGGAACATCGTGAAAGC-3′
	5′-TGGAGGTGCACAGAGCTGATA-3′
TGF-β	5′-CGCAACAACGCCATCTATGA-3′
	5′-ACCAAGGTAACGCCAGGAAT-3′
Fibronectin	5′-ATGATGAGGTGCACGTGTGT-3′
	5′-TCTCCCAGGAGTCACCAATC-3′
β-actin	5′-AGAGGGAAATCGTGCGTGAC-3′
	5′-CAATAGTGATGACCTGGCCGT-3′
Collagen-1	5′-GTCCTAGTCGATGGCTGCTC-3′
	5′-CAATGTCCAGAGGTGCAATG-3′

Table S2. Baseline characteristics of IgAN patients according to renal TLS

	Without TLO	With TLOs	P value
Number	27	30	
Age, years	33.48 ± 8.00	40.77 ± 11.39	0.008
Sex (Male, %)	10, 43.48%	13, 56.52%	0.788
Hemoglobin, g/L	132.79 ± 13.28	120.60 ± 20.38	0.017
Albumin, g/L	42.05 ± 4.89	38.32 ± 6.28	0.016
Total cholesterol (mmol/L)	4.53 ± 1.37	4.99 ± 1.34	0.201
Proteinuria, g/d	0.77 ± 0.52	2.20 ± 2.07	0.002
Serum creatinine (μmol/L)	76.22 ± 26.32	127.87 ± 71.39	<0.001
Blood urea nitrogen (mmol/L)	5.03 ± 1.44	7.06 ± 2.97	0.002
Uric acid, μmol/L	330.79 ± 79.60	391.55 ± 124.74	0.035
eGFR, ml/min/1.73m^2	102.31 ± 23.53	66.75 ± 31.41	<0.001

TLS, tertiary lymphoid structure; eGFR, estimated glomerular rate.

Table S3. Detailed information about immunotherapy in 10 IgAN patients.

	Corticosteroids	Cyclophosphamide	Tacrolimus
Patient 1	√		
Patient 2	√		
Patient 3	√		
Patient 4	√		
Patient 5	√	√	
Patient 6	√		
Patient 7	√	√	
Patient 8	√		√
Patient 9	√		√
Patient 10	√		

Table S4. Characteristics of 10 IgAN patients before and after immunotherapy treatment for 8-12 weeks.

	before	after	P value
Age, years	40.20 ± 11.63		-
Sex (Male, %)	6,60%		-
Albumin, g/L	36.69 ± 10.60	36.05 ± 6.18	0.068
Total cholesterol (mmol/L)	5.29 ± 1.49	4.89 ± 1.50	0.014
Proteinuria, g/d	3.52 ± 3.04	1.25 ± 1.51	0.016
Serum creatinine (μmol/L)	137.00 ± 85.10	99.4 ± 43.83	0.145
Blood urea nitrogen (mmol/L)	8.21 ± 4.64	5.87 ± 2.02	0.039
Uric acid, μmol/L	475.81 ± 144.12	349.60 ± 77.01	0.060
eGFR, ml/min/1.73m^2	65.67 ± 34.60	83.88 ± 32.39	0.906

eGFR, estimated glomerular rate

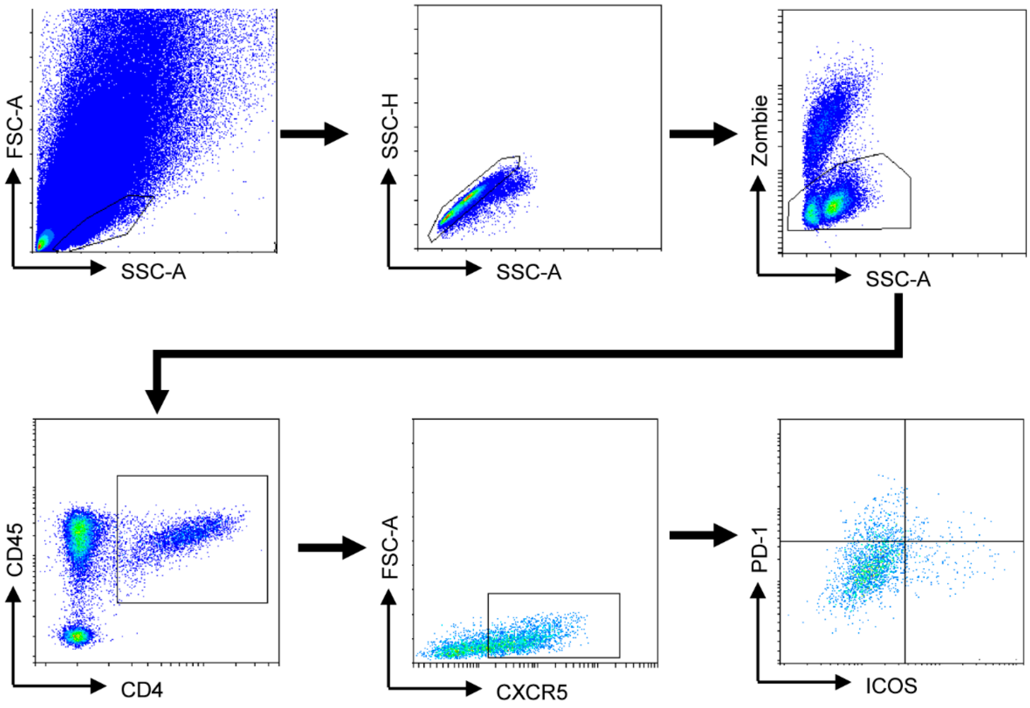

Figure S1. Scheme of cell sorting for TFH.

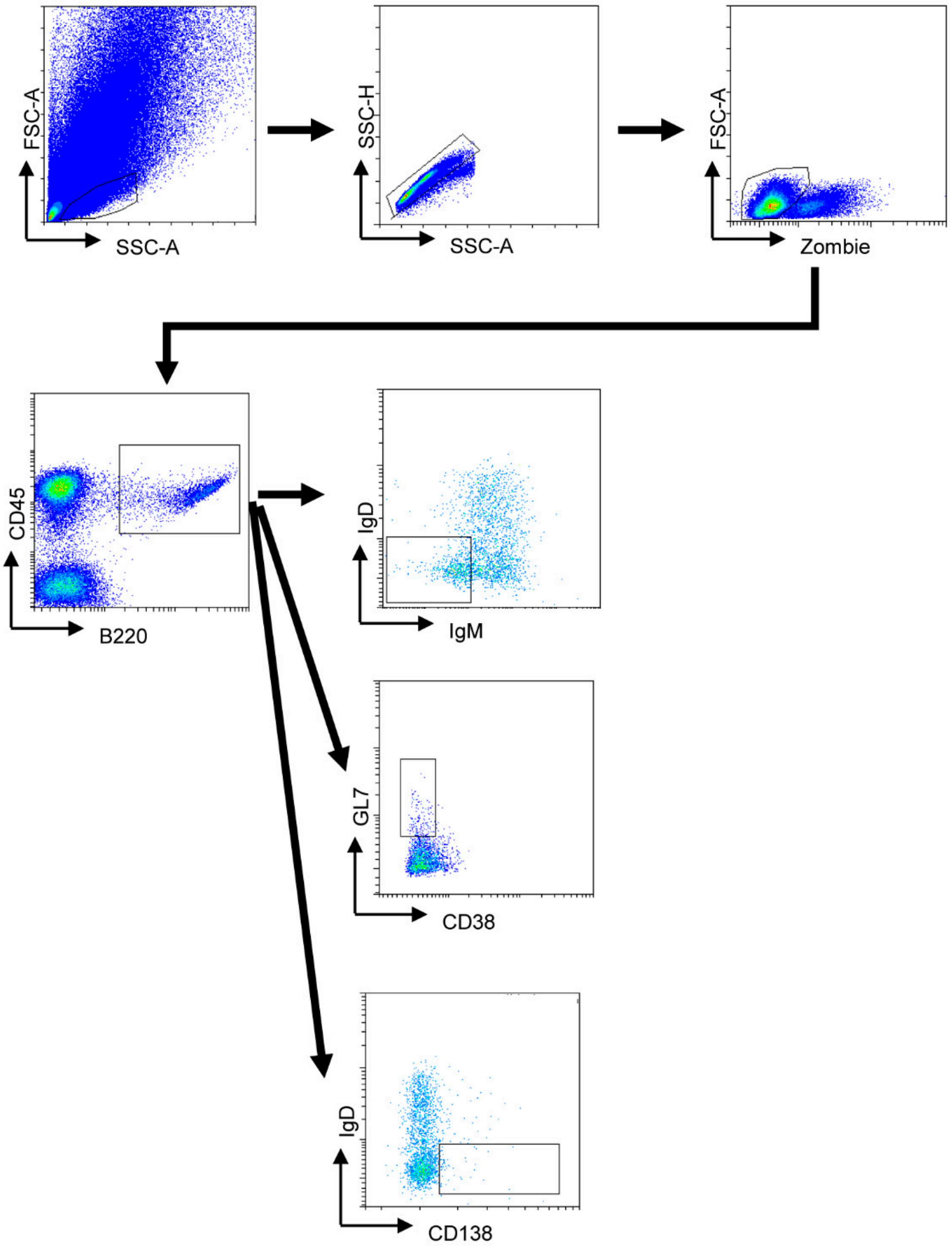

Figure S2. Scheme of cell sorting for B lymphocyte subsets.
